# Optimizing MobileNetV2 for improved accuracy in early gastric cancer detection based on dynamic pelican optimizer

**DOI:** 10.1016/j.heliyon.2024.e35854

**Published:** 2024-08-06

**Authors:** Guoping Zhou, Qiyu He, Xiaoli Liu, Xinghua Kai, Weikang Cao, Junning Ding, Bufeng Zhuang, Shuhua Xu, Myo Thwin

**Affiliations:** a Department of General Surgery, Dongtai Hospital of Traditional Chinese Medicine, Dongtai, 224221, Jiangsu, China; b Department of Thoracic Surgery, Dongtai Hospital of Traditional Chinese Medicine, Dongtai, 224221, Jiangsu, China; c Department of Pathology, Dongtai Hospital of Traditional Chinese Medicine, Dongtai, 224221, Jiangsu, China; d Department of Thoracic Surgery, Shanghai Ninth People's Hospital, Shanghai, 201900, Shanghai, China; e Yangon Technological University, Myanmar; f College of Technical Engineering, The Islamic University, Najaf, Iraq

**Keywords:** Early diagnosis, Gastric cancer, MobileNetV2, Enhanced pelican optimization algorithm, Deep learning, Convolutional neural networks, Endoscopists

## Abstract

This paper presents an innovative framework for the automated diagnosis of gastric cancer using artificial intelligence. The proposed approach utilizes a customized deep learning model called MobileNetV2, which is optimized using a Dynamic variant of the Pelican Optimization Algorithm (DPOA). By combining these advanced techniques, it is feasible to achieve highly accurate results when applied to a dataset of endoscopic gastric images. To evaluate the performance of the model based on the benchmark, its data is divided into training (80 %) and testing (20 %) sets. The MobileNetV2/DPOA model demonstrated an impressive accuracy of 97.73 %, precision of 97.88 %, specificity of 97.72 %, sensitivity of 96.35 %, Matthews Correlation Coefficient (MCC) of 96.58 %, and F1-score of 98.41 %. These results surpassed those obtained by other well-known models, such as Convolutional Neural Networks (CNN), Mask Region-Based Convolutional Neural Networks (Mask R–CNN), U-Net, Deep Stacked Sparse Autoencoder Neural Networks (SANNs), and DeepLab v3+, in terms of most quantitative metrics. Despite the promising outcomes, it is important to note that further research is needed. Specifically, larger and more diverse datasets as well as exhaustive clinical validation are necessary to validate the effectiveness of the proposed method. By implementing this innovative approach in the detection of gastric cancer, it is possible to enhance the speed and accuracy of diagnosis, leading to improved patient care and better allocation of healthcare resources.

## 1 Introduction

Gastric cancer, also known as stomach cancer, is a malignant disease that originates in the cells lining the stomach. It is a significant global health concern and is one of the leading causes of cancer-related deaths worldwide. The disease progresses slowly over several years, and in its early stages, it often presents with little to no symptoms or nonspecific signs, making it challenging to diagnose. However, as the disease advances, symptoms, such as persistent indigestion, abdominal discomfort, unintentional weight loss, loss of appetite, nausea, and vomiting, may arise [1].

Early diagnosis is crucial in the effective management of gastric cancer. Detecting the disease at an early stage significantly increases the chances of successful treatment and improves patient outcomes. Early-stage gastric cancer is typically confined to the innermost layers of the stomach wall, making it more amenable to curative surgeries. Additionally, early diagnosis allows healthcare professionals to implement less aggressive treatment approaches, reducing the need for extensive surgery, chemotherapy, and radiation therapy [2].

Early diagnosis of gastric cancer not only improves individual patient outcomes but also has broader implications for healthcare systems. Timely detection allows for better resource allocation, as patients that have been diagnosed at earlier stages generally require less intensive and costly treatments than those diagnosed at advanced stages. Moreover, early diagnosis may reduce the various diagnostic methods that are employed to achieve early diagnosis, with esophagogastroduodenoscopy (EGD) being the gold standard. EGD involves inserting a thin, flexible tube equipped with a light and a camera into the patient's mouth and down the esophagus to examine the stomach and duodenum. In recent years, there has been a growing emphasis on using advanced technologies and techniques to enhance the accuracy and efficiency of gastric cancer diagnosis.

Li et al. [3] developed a novel system based on a Convolutional Neural Network (CNN) to analyze gastric mucosal lesions observed through Magnifying endoscopy with Narrow Band Imaging (M-NBI). The CNN model (Inception-v3) was trained and established using a total of 386 images of non-cancerous lesions and 1702 images of early gastric cancer [4]. The diagnostic capabilities of the CNN system were evaluated using 341 endoscopic images, and the results demonstrated a sensitivity, specificity, and accuracy of 91.18 %, 90.64 %, and 90.91 %, respectively in diagnosing early gastric cancer. The CNN system exhibited superior diagnostic sensitivity compared to human experts and non-experts, with no significant difference in specificity and accuracy. These findings suggested that the CNN system could serve as a valuable tool in the diagnosis of early gastric cancer. However, limitations of this work might include the restricted number of images used for training and evaluation, as well as potential challenges in generalizing the results to different patient populations or clinical settings. Further optimization and development of the CNN diagnostic system are necessary to enhance its performance in the medical field.

Shibata et al. [5] proposed a novel approach for the automated detection and segmentation of early gastric cancerous regions from gastrointestinal endoscopic images utilizing Mask R–CNN. A total of 1208 healthy and 533 cancerous images were collected for the study. The Mask R–CNN model was employed to detect and segment the gastric cancerous region in the endoscopic images, resulting in a bounding box and a label image. The performance evaluation, utilizing five-fold cross-validation, demonstrated a sensitivity of 96.0 % and an average of 0.10 false positives per image. In terms of segmentation, the average Dice index was 71 %. These findings suggested that the proposed scheme had the potential to detect gastric cancer and evaluate the invasive region during gastrointestinal endoscopy. However, limitations of this work might include the limited sample size and the need for further validation using larger and diverse datasets. Additionally, the performance of the method in real-world clinical settings should be assessed before its widespread application.

Teramoto et al. [6] introduced an object detection model called U-Net R–CNN to propose an automated detection method for early gastric cancer from endoscopic images. The U-Net R–CNN model was based on a semantic segmentation technique that performed local analysis to extract target objects. Early candidates for gastric cancer were detected using the U-Net model for semantic segmentation and then classified as gastric cancerous cases or false positives using a convolutional neural network. The detection performance was evaluated using a 5-fold cross-validation method with 1208 healthy subject images and 533 gastric cancerous images. The use of DenseNet169 for box classification resulted in a detection sensitivity of 98 % and a false positive rate of 0.01 per image, indicating improved performance compared to previous methods. The proposed method held promise for the automated detection of early gastric cancer from endoscopic images. However, limitations of this work included the need for further validation with larger and more diverse datasets, as well as evaluation of its performance in real-world clinical settings. Additionally, the generalizability of the proposed method should be assessed across different populations and imaging conditions.

Aslam et al. [7] proposed a novel approach for the identification of early-stage gastric cancer utilizing deep learning techniques. The investigators developed a Computer-Aided Diagnosis (CAD) system that employed a stacked sparse autoencoder for feature extraction from unlabeled breath samples. A Softmax classifier was integrated to classify gastric cancer based on the extracted features. The CAD system was successful in distinguishing between early gastric cancer, advanced gastric cancer, and healthy individuals by identifying fifty peaks in each spectrum. The deep stacked sparse autoencoder neural network architecture achieved a high overall accuracy of 98.7 % for advanced gastric cancer classification and 97.3 % for early gastric cancer detection. Furthermore, the model demonstrated good results for clinical application. However, limitations of this study may include the requirement for further validation with larger and more diverse datasets, as well as evaluation in real-world clinical settings to assess its performance in practical scenarios. Additionally, the generalizability of the proposed CAD system should be examined across different populations and breath collection techniques.

Wang et al. [8] proposed an automatic segmentation model for gastric cancer based on the Deeplab v3+ neural network. The purpose was to enhance the efficiency of recognizing and segmenting cancerous regions in gastric cancerous pathological slice images. A comparison was made with the SegNet and ICNet models, utilizing 1240 gastric cancerous pathological slice images to assess sensitivity, specificity, accuracy, and Dice coefficient. The results revealed that Deeplab v3+ achieved a sensitivity of 91.45 %, specificity of 92.31 %, accuracy of 95.76 %, and a Dice coefficient of 91.66 %, surpassing the other models by more than 12 %. Moreover, the model demonstrated a reduction in parameter scale. Overall, the proposed model that was based on Deeplab v3+ successfully improved segmentation accuracy and conserved computing resources, making it valuable for medical image analysis and gastric cancer diagnosis. However, limitations of this work included the need for further evaluation and validation using larger and more diverse datasets to assess performance across various clinical scenarios. The generalizability of the proposed segmentation model should be studied in different populations and types of gastric cancerous pathology. Additionally, further research was necessary to explore the model's applicability in real-world clinical settings to determine its practical effectiveness and reliability.

These cutting-edge techniques enable the identification of subtle visual cues and patterns that may indicate the presence of gastric cancer at early stages, even when they are not apparent to the human eye.

Despite the growing interest in AI-augmented diagnostics, there are several obstacles that hinder the full realization of its potential. One of these challenges is the complexity of medical image data. Medical images can vary significantly in terms of modality, resolution, dimensionality, and intrinsic properties. This variability creates inherent complications when trying to develop AI models that can be universally applied across different types of medical images.

Another obstacle is the issue of class imbalance in many medical datasets. Skewed class distributions can have a negative impact on the performance and generalizability of supervised learning algorithms. This imbalance can make it difficult for AI models to accurately classify and predict outcomes.

Furthermore, the interpretation of “black-box” models poses a challenge in the field of AI-augmented diagnostics. Deep learning algorithms, in particular, struggle to provide actionable insights and explanations for their predictions. This lack of interpretability hinders the acceptance of these models among medical professionals who require a clear rationale behind the predictions.

Additionally, there is a need for sufficient clinical validation of AI models. While promising results may be achieved at the laboratory level, it is crucial to validate and adapt these models to real-world constraints. The translation of laboratory-level performances to clinical reality requires meticulous validation to ensure the reliability and effectiveness of AI-augmented diagnostics.

In an effort to address some of these challenges, the study proposes an innovative AI-driven framework for gastric cancer detection. This framework utilizes a customized deep learning paradigm called MobileNetV2, combined with a Dynamic evolution of the Pelican Optimization Algorithm (DPOA). By using these techniques, it is an attempt to overcome the complexities of medical image data, improve the performance of supervised learning algorithms, enhance interpretability of AI models, and ensure sufficient clinical validation for real-world application.

By combining MobileNetV2, a branch of deep learning, with the Enhanced Pelican optimization algorithm, the researchers aimed to improve the detection of gastric cancer from EGD images. The integration of the MobileNetV2 model into the diagnostic process offers several advantages. These algorithms can rapidly analyze a large number of EGD images, accurately detecting suspicious areas and aiding in the early identification of potential malignancies. By enhancing the accuracy and efficiency of diagnosis, such methodologies hold promise in reducing missed or delayed diagnoses, thus enabling timely initiation of appropriate treatment strategies.

However, the research can face some limitations. The study focused only on a single type of gastric cancer, which limits its immediate applicability to other digestive tract neoplasms. To enhance the diagnostic understanding of MobileNetV2/DPOA, it is important to explore multiple cancer types. Relying exclusively on MobileNetV2/DPOA leaves room for investigating ensemble methods or hybrid architectures to improve the predictive capacity. The lack of clinical validation overlooks real-world clinical factors, such as illumination variations and specimen preparation irregularities. Bridging this gap through collaboration with medical institutions is essential. The absence of external testing hampers the definitive confirmation of the model's effectiveness and replicability. Prospective trials and partnerships with other centers can provide insights beyond the experimental setting. The computational demands and expertise required for advanced ML techniques present challenges. Enhancing accessibility to AI-driven diagnostic tools can be achieved by developing user-friendly platforms and utilizing cloud services. Lastly, addressing ethical considerations, such as privacy, security, informed consent, and liability, is crucial for fostering trust and responsible innovation in AI-supported healthcare applications.

## 2 Dataset description

The dataset utilized in this study comprises Endoscopic images of gastric cancer obtained from 42 healthy subjects and 95 cases of gastric cancer. The images were acquired during preoperative examinations conducted at Fujita Health University Hospital between July 16, 2013, and August 30, 2017, using Whole-Slide Imaging (WSI) technology and stored in the PNG format [9]. This dataset can be achieved by mailing to: *jgca@ koto. kpu-m. ac. jp*.

The dataset is classified into four primary groups, namely 895 NT (Non-Tumorous) images, 1292 TA (Tubular Adenoma) images, 1495 MA (Mucosal Adenocarcinoma) images, and 608 PA (Papillary Adenocarcinoma) images. To ensure precise evaluation, the original dataset was randomly divided into two sets, including a training set and a test set, with the training set comprising 80 % of the dataset and the test set containing the remaining 20 %.

Regarding the distribution of gastric carcinoma cases within the dataset, the following observations were made. Initially, 9 cases were found in the upper third of the stomach, 52 in the middle third, and 33 in the lower third. The majority of cases (63) were classified as Type 0-Ilc, indicating a specific macroscopic appearance of the tumor. Additionally, there were 21 cases of mixed Type 0 tumors, which combined different macroscopic types. The dataset included 71 cases classified as T1a, indicating limited tumor invasion confined to the mucosa, and 23 cases classified as T1b, representing submucosal invasion. Based on histopathological analysis, 75 cases were classified as differentiated, indicating well-defined tumor tissue characteristics. Additionally, there were 13 cases of undifferentiated tumors and 6 cases that exhibited mixed features, combining differentiated and undifferentiated components.

**Fig. 1:**
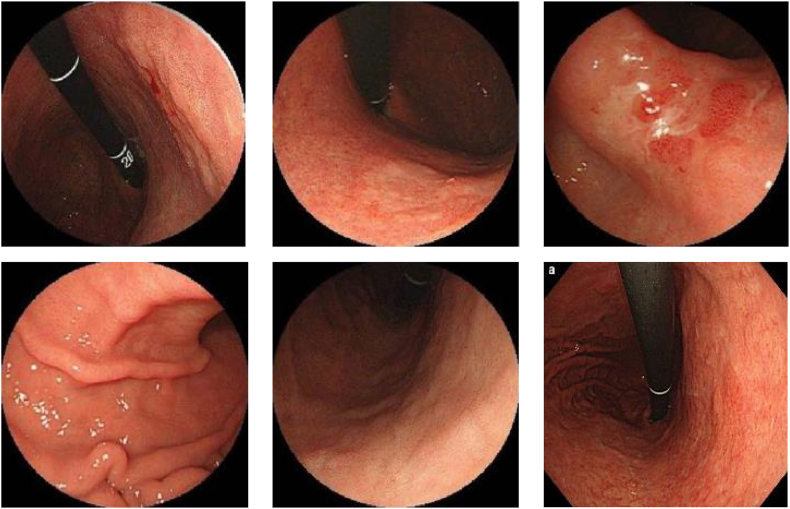
Some samples of the gastric cancer from the dataset.

Fig. (1) shows some samples of the gastric images from the dataset.

These findings highlight the visual diversity and heterogeneity of gastric cancer cases captured in the dataset, emphasizing the importance of considering various tumor characteristics for accurate analysis and evaluation.

## 3 Preprocessing

### 3.1 Noise reduction

Noise reduction in gastric endoscopic images is of utmost importance due to its significant impact on image quality and diagnostic accuracy. Gastric endoscopic images are critical tools for detecting and evaluating various gastrointestinal conditions, including gastritis, gastric cancer, polyps, and ulcers. However, these images are often affected by noise, which can obscure important details and compromise the visibility and interpretation of pathological findings. By applying noise reduction techniques, the clarity and definition of the images are significantly improved. This enables medical professionals to discern subtle lesions, identify fine structures, and assess tissue texture with greater precision. With reduced noise, the accuracy of diagnosis is significantly enhanced, minimizing the risk of misinterpretation or missed detection of potential abnormalities.

Moreover, noise reduction facilitates more accurate measurements and evaluations, enabling precise treatment planning. This is particularly important when determining the size, shape, and location of lesions or abnormalities within the stomach, as it allows for appropriate intervention strategies, such as targeted biopsies, endoscopic resections, or surgical procedures. Additionally, noise-free images provide a reliable baseline for follow-up examinations, facilitating efficient monitoring of treatment response and disease progression. Comparisons between noise-free images taken at different time points ensure accurate assessment and identification of potential recurrence.

The utilization of Non-Local Means (NLM) for this purpose has been found to be highly advantageous. NLM is a versatile denoising technique that effectively minimizes noise while preserving the crucial structures within these medical images. The process commences by extracting small patches from the image, typically in the form of square regions, containing pixel intensities. These patches are then compared to determine their similarity using metrics, such as Euclidean distance or structural similarity index. Based on these similarities, weights are calculated for each patch to indicate their contributions to the denoising process. The filtering step utilizes these weights to compute a weighted average of pixel values in neighboring patches, effectively reducing noise while retaining vital information. The NLM can be mathematically defined as follows:

The first step in NLM is to extract a patch of size N×N pixels centered around each pixel in the input image. This can be represented mathematically as Eq. (1).

(1) P(i)=[p(j):j∈Ω(i)]

where, P(i) is the patch that is centered around pixel i, p(j) is the value of the pixel that is located at location j, and (i) is the neighborhood of i, which consists of pixels that are within a certain distance of i.

After that, a search window that is W×W pixels in size is constructed around each patch P(i) in order to locate other patches inside the picture that are comparable to it. Eq. (2) is a mathematical representation of this:

(2) S(i)=[P(j):j∈Ψ(i)]

where, S(i) is the search window that is centered around patch i, P(j) is the patch that is centered around pixel j, and (i) is the neighborhood that is centered around patch i and consists of patches that are within a certain distance of P(i).

When the search window has been established, the next step is to determine the degree of similarity that exists between each patch P(i) and the patches that surround it. The Gaussian weighted Euclidean distance function is one method that may be used to assess similarity that can be defined as Eq. (3).

(3) $$d\left(P\left(i\right),P\left(j\right)\right)=\exp\left(\left(-\frac{1}{2h^{2}}\right)\times {\left|\left|P\left(i\right)-\;P\left(j\right)\right|\right|}^{2}\right)$$

where, h is a filtering parameter, P(i) and P(j) denote two patches, and the Euclidean distance between the patches is denoted by the notation ||.||.

On the basis of the similarity measure, a weight w(i,j) is computed for every patch j that is included in the search window S(i) as Eq. (4).

(4) $$w\left(i,j\right)=\exp\left(\left(-\frac{1}{2h^{2}}\right)\times {\left|\left|P\left(i\right)-P\left(j\right)\right|\right|}^{2}\right)$$

The significance of patch j’s input to the denoising process is represented by the weight w(i,j). Patches that are less similar to the current patch have lower weights, and patches that are more similar to the current patch have higher weights.

At last, the value of each pixel in the image that has been denoised is computed as a weighted average of the values of its neighboring pixels, using the weights that is defined in Eq. (5).

(5) $$v_{denoised}\left(i\right)=\left(\frac{1}{W_{p}}\right)\sum \left(j\in S\left(i\right)\right)w\left(i,j\right)v\left(j\right)$$

where, $v_{denoised}\left(i\right)$ represents the denoised value of pixel i, and $W_{p}$ denotes the sum of all of the weights that are included inside the search window. S(i) represents the search window, w(i,j) represents the weight that is allocated to patch j in the window, and v(j) represents the pixel value of patch j.

**Fig. 2:**
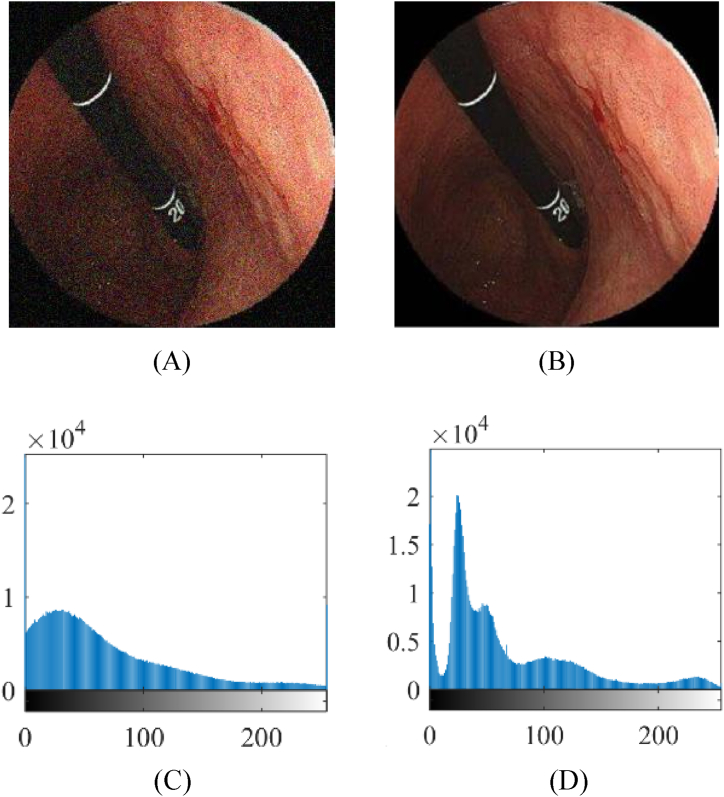
Results of the gastric images noise reduction based on NLM, including (A) original image, (B) processed image, (C) histogram of (A), and (D) histogram of (B).

Fig. (2) demonstrates the application of Non-Local Means (NLM) for noise reduction in gastric images.

As can be observed from Fig. (2), figure (A) shows the original image, while figure (B) displays the image after being processed with NLM. Figure (C) depicts the histogram of the original image, and figure (D) illustrates the histogram of the processed image.

The NLM is a powerful technique for reducing noise in gastric endoscopic images, improving image quality and enabling accurate diagnosis of gastrointestinal conditions like gastritis, ulcers, and gastric cancer. It can handle various types of noise, including Gaussian, impulse, and speckle noise, and is less sensitive to parameter tuning compared to other denoising methods. However, its computational demands can be significant, especially for larger images or real-time applications. Efficient implementation and optimization techniques are used to make NLM denoising more practical and accessible.

### 3.2 CLAHE*-*based Contrast enhancement

The CLAHE stands for Contrast Limited Adaptive Histogram Equalization, an image processing technique utilized to enhance the contrast in images. The CLAHE is an extension of the conventional histogram equalization method, incorporating adaptive and limited contrast enhancement. Unlike traditional histogram equalization, which equalizes the overall image histogram, CLAHE partitions the image into small regions referred to as tiles or blocks. For each tile, a separate histogram is computed, and the cumulative distribution function (CDF) of the histogram is applied to enhance the contrast within that particular tile.

To prevent over-amplification of noise and extreme contrast enhancement, CLAHE applies a contrast limit that sets a threshold, beyond which contrast enhancement is limited. This constraint ensures that the enhancement remains within a specified range and prevents artifacts or unnatural amplification. By applying contrast enhancement locally on smaller tiles, CLAHE is able to adaptively enhance the contrast based on the local characteristics of the image. It helps to overcome the limitations of traditional histogram equalization, which tends to amplify noise and result in uneven enhancement across different regions of the image.

CLAHE can be applied to enhance the contrast of gastric endoscopic images, providing improved visibility and detail. The process begins by preprocessing the image, which may involve resizing and noise reduction to prepare it for contrast enhancement. Next, the image is divided into small, overlapping tiles of equal size. The size of tile depends on the specific image characteristics and desired level of detail, typically ranging from 32×32 to 128×128 pixels. This tiling allows for local analysis and enhancement of contrast in different parts of the image. Now, histogram equalization is applied individually to each tile. This process redistributes the pixel intensities in a way that enhances overall contrast. However, traditional histogram equalization techniques may amplify noise and lead to over-enhancement that is where CLAHE plays a crucial role.

CLAHE addresses these issues by applying adaptive and limited contrast enhancement. Within each tile, the histogram is clipped at a predefined contrast limit, preventing from excessive augmentation. After clipping, the histogram is equalized using the cumulative distribution function, resulting in enhanced contrast while avoiding artifacts or unnatural amplification. After applying CLAHE to all tiles, the enhanced tiles are combined to reconstruct the final image. This can be achieved through blending techniques, such as overlapping, averaging, or interpolation. The goal is to create a seamless transition between the tiles, ensuring a visually coherent and enhanced image.

**Fig. 3:**
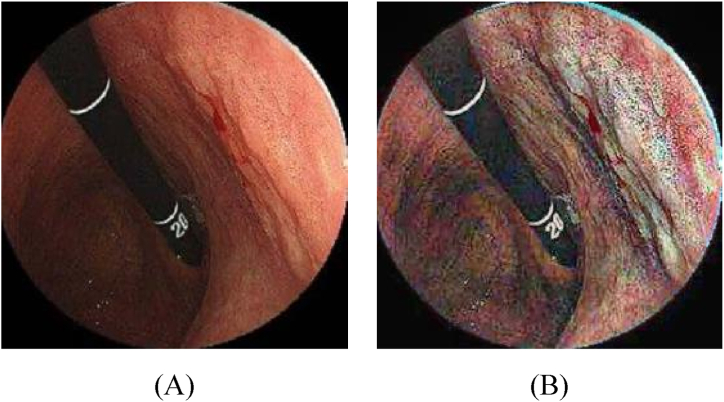
An instance of contrast enhancement applied to gastric images: (A) Image before noise reduction, and (B) the image after contrast enhancement.

Fig. (3) shows an instance of contrast enhancement applied to gastric images. The first step involved removing noise from the original image.

The resulting denoised image is shown in figure (A). To enhance the contrast of this image, a contrast enhancement technique was applied, and the resulting image is shown in figure (B).

### 3.3 Data augmentation

Owing to the restricted accessibility of the pathological images, augmentation technique has been utilized to amplify the volume of pathological data.

In this study, data augmentation is utilized to enhance the gastric images in pathological image dataset. In particular, the dataset is diversified using various augmentation methods, including random rotation, random X and Y-reflection, random X and Y-scale, random X and Y-translation, and random X and Y-shear. These methods were selected to improve the robustness and generalization of the model by introducing variability in the dataset [10]. Specifically, random rotation was employed to rotate the image in a random angle, whereas random X and Y-shear were used to distort the image along the respective axes. Random X and Y-reflections were applied to horizontally and vertically flip the image, respectively. Random X and Y-scale allowed for non-uniform scaling along the respective axes. Finally, random X and Y-translation were used to shift the image in both directions. Together, these augmentation techniques resulted in an expanded dataset with increased variability, which was beneficial for training deep learning models to accurately classify gastric diseases.

The data augmentation techniques used in this study involved various transformations applied to the gastric images in pathological images. Random rotation was employed to randomly rotate the images within a range of −100 to 100°. This allowed the model to learn from gastric images with different orientations, improving its ability to recognize and classify gastric cancer regardless of their rotational position. Random X and Y-shear involved skewing the image along the X and Y axes. By applying random shears within the range of −0.04 to 0.04, distortions similar to those caused by patient positioning or scanner artifacts could be simulated. This helped the model become more robust to such variations.

Random X and Y-reflection randomly flipped the image horizontally or vertically. This provided additional examples of gastric images from different viewpoints, enabling the model to generalize well to various spatial orientations. Random X and Y-translation involved randomly shifting the images horizontally and vertically within the range of −60 to 60 pixels. This simulated shifts in the position of the gastric region within the pathological images, enhancing the model's ability to detect and classify gastric images with varying locations. Scaling was done by resizing the image while preserving its aspect ratio. Random X and Y scales, which were within the range of 0.6–5, were applied to simulate differences in gastric sizes present in the pathological images. This allowed the model to learn how to recognize cancer of various dimensions.

**Fig. 4:**
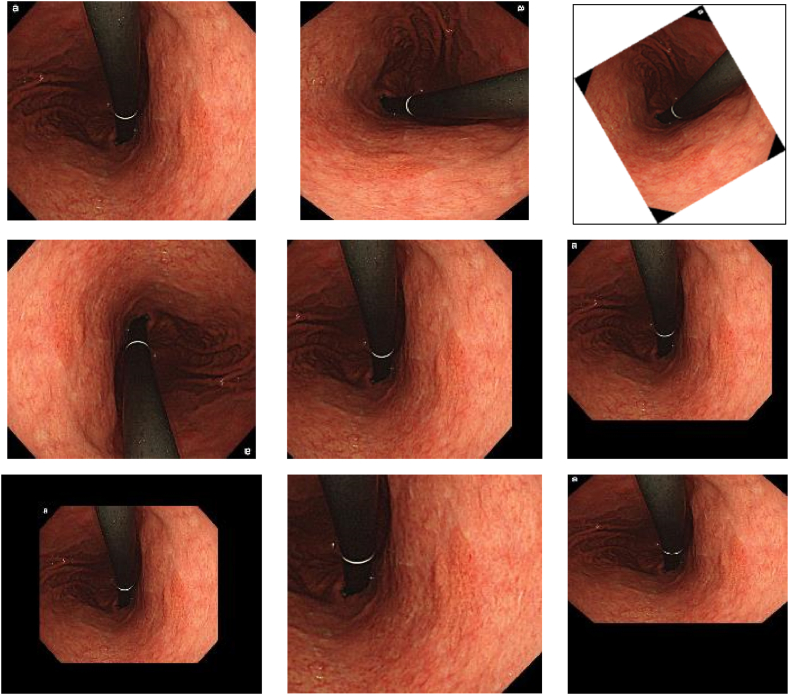
Sequence of illustrative pathological slice images demonstrating the practical application of data augmentation methods on the gastric images.

Fig. (4) depicts a sequence of illustrative pathological slice images demonstrating the practical application of data augmentation methods on the gastric images.

The series of images is expected to commence with the unmodified gastric image, which serves as the fundamental reference for comparative analysis. The subsequent images in the sequence would showcase the impact of each data augmentation technique in isolation.

## 4 Model and configuration of the MobileNetV2

### 4.1 MobileNetV2

**Fig. 5:**
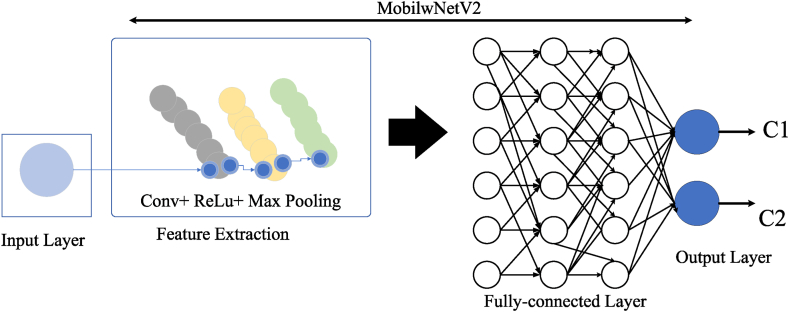
The configuration of the MN-V2.

Deep learning is a sophisticated artificial intelligence method that employs neural networks comprising multiple layers to acquire knowledge from extensive data and detect intricate patterns [11,12]. MobileNetV2, on the other hand, is a streamlined neural network design specifically tailored for mobile devices. It utilizes an inverted residual structure to process images with utmost precision and minimal delay. A group of MobileNetV2 (MN-V2) has been recommended in the current investigation to solve the issue of colposcopy image groups. The reason for choosing MN-V2 is due to its structure and inspiration [13]. However, it has been observed that using optical identification in Net training results in overfitting, particularly when dealing with a small dataset. By utilizing a more compact and communicative network called MN-V2, it was proven that the aforementioned effect is not valid. The main feature of MN-V2 is to optimize memory usage and implementation speed at a low cost once an error occurs. Simplification of parameter tuning and experimentation is achieved through higher implementation speed. In order to achieve minimal memory usage, it is preferable to have a network structure that is designed accordingly. There are two serious consequences, namely IR (Inverted Residual) and SD-WC (Separable Depth-Wise Convolution), to elucidate the formation of MN-V2. The concepts, as mentioned above, will be given below. Fig. (5) demonstrates the organization of the MN-V2.

The SD-WC, Xception, MN-V2, and ShuffleNet, have employed some complementary productive simulations. The SD-WC has replaced the typical obstacle through two methods. The first operator employs a convolution plan-wise, which generates a unique complexity for every belonging plan. The plans of the property have been arranged and loaded by utilizing a point-wise convolution in the second operation with a 1×1 kernel. Using this operation, the entire property strategies were organized simultaneously that resulted in a quick and efficient organization of the image across the network's width, height, and size. While the SD-WC moves the image horizontally and vertically during the first procedure, the second operation adjusts the picture size of the network. The calculated value for normal convolutions and the SD-WC can be determined using Eqs. (6), (7).

(6) $$Z_{N}=d_{j}.\;h_{j}.\;w_{j}.\;K^{2}$$

(7) $$Z_{s}=d_{j}\left(k^{2}+d_{i}\right).\;h_{j}.w_{j}$$

The conventional convolutions and the associated value of SD-WC are represented by $Z_{N}$ and $Z_{s}$, respectively. The input and output layers are denoted by i and j, where $d_{i}$ and $d_{j}$ represent the number of output and input property plans. The height and width of the input maps are illustrated by $h_{j}$ and $w_{j}$. Lastly, the filter size is determined by k.

The benefits of utilizing SD-WC over regular convolutions can be articulated based on Eq. (8).

(8) $$\frac{Z_{N}}{Z_{s}}=\frac{k^{2}.\;d_{i}}{k^{2}+d_{i}}$$

Necessary units are contained in the blocks that are overturned and remaining. Three regular operators have been employed along with blocks and other relationships within these blocks. The first and last operators use 1×1 filters that shift the input layer to the central layer and from the central layer to the output layer. 3×3 filters have been used to facilitate the analysis of the layers in the middle. There are numerous networks in the blocks that remain, particularly in the first and last convolutions, compared to the internal convolution blocks.

There are fewer networks in the first and last convolutions in the overturned remaining. Fewer connections exist between the property plans of the MN-V2 model compared to the ResNet model, leading to a reduced number of connecting attitudes between the first and latter networks. As two simulations load numerous sections, the sizes of the layers change significantly. The remaining connection clearance undergoes significant modifications, which enables the MN-V2 to make beneficial use of memory during its creation.

The literature has documented the use of the MN-V2 net information, which was made available with unrestricted base code on GitHub. To address the concerns raised in this study, a substantial alteration will be made to the network structure, which will be clearly explained. The objective of this modification is to ensure that the network is in line with the relevant issue.

### 4.2 MobileNetV2 (MN-V2) tuning

The MN-V2 network comprises a collection of well-ordered blocks that have been flipped over. These blocks are organized into two convolutions that act as connectors, transferring input from the input layer to the central layer and from the central layer to the output layer [14]. The convolution's fundamental output is determined by two layers, known as global mean pooling and inference. The MN-V2 network is primarily designed for ImageNet classification, which includes one thousand categories. As a result, the final convolution layer is larger, with 1280 network property plans and a filter with a dimension of 1×1.

The classification of cervical lesions is confined to just four categories, enabling high accuracy with minimal image representation, regardless of whether single or multiple samples are used. The last layer of the convolutional block, following the inverted block, has been modified to limit the output size to either 32 or 64 networks rather than 1280. If the layer has been employed in the gathering, it will match the final layer. The connection of the acquired property plans and other imitations is facilitated by the plural net. When targeting this layer, the methods undergo global mean pooling, resulting in a 32-64-part vector if a specific model is used. The vector is then controlled by layers that are fully connected to compute the outcomes. To preserve previously learned variables, additional layers remained unchanged while the main model of MN-V2 was trained on ImageNet. During the teaching process, the entire network undergoes fine-tuning for the aforementioned reasons.

### 4.3 Loss function

It is possible for the model to be trained to favor certain classes over others due to an imbalanced dataset, which could cause harm. This is because the model may encounter ambiguous examples that could belong to multiple classes during training, leading to updates in the simulation parameters that favor one direction [15]. To address this issue, a model is created with a focus on the negative aspects of the training process, which can be achieved through the use of the loss function. The default loss function used in training the model is cross-entropy loss, which is explained in Eq. (9).

(9) $$M_{y,z}=-\sum_{j}^{N}\vec{Z_{j}}\ln\left(V\left(\vec{y_{j}}\right)\right)$$

The illustration of cross-entropy loss is shown using $M_{y,z}$, while N and i are used to demonstrate the number of instances and training, respectively. The input tensors of $\vec{y}$ and $\vec{z\;}$, along with 1hot-vectors that identify the final outcome of the network, are provided. $V(\;\vec{y_{j})}$ close paren is a function that results in a four-dimensional vector with $\vec{y_{j}}$ representing the input selected in each category. Finally, by performing an onward pass within the network $\vec{y_{j}\;}$ and utilizing Softmax, the achievement has been attained.

The weighted cross entropy loss operation utilizes the weight vector $\vec{w\;}$ to enhance the importance of multiple categories. Its purpose is to increase the significance of various categories. In the focal loss function, an element is used to simplify the more straightforward examples, which is denoted by ${\left(1-V\left(\vec{y_{j}}\;\right)\right)}^{\alpha }$ then converted to the delta. The two functions mentioned above are expressed in Eqs. (10), (11).

(10) $$WM_{\vec{y,}\vec{z},\vec{w}}=-\sum_{J}^{N}\vec{z_{j}}\;\left[\vec{W}⊙\ln\left(V\left(\vec{y_{j}}\right)\right)\right]$$

(11) $$R_{\vec{z},\vec{y}}=-\sum_{J}^{N}\vec{z_{j}}\left[{1-V\left(\vec{y_{j}}\;\right))}^{\alpha }⊙\ln\ln\left(V\left(\vec{y_{j}}\right)\right)\right]$$

The weighted cross entropy loss function is illustrated using $WM_{\vec{z},\vec{w},\vec{y}}$, end subscript. The vector of category weight, denoted by $\vec{w}$, has four dimensions and is used to calculate the outcome using ⨀, which represents component-wise multiplication between two vectors. The resulting vector has sizes that are comparable to the components it generates. The focal function is demonstrated by $R_{\vec{z},\vec{y}}$, while delta is a component that adds the loss for simpler cases. A tool is used in the loss function to measure class imbalances, which is called the simple cross entropy ($M_{\vec{y},\vec{z}})$. Currently, the net receives an unequal proportion of the overall example due to class imbalances.

When employing ${\vec{y}}_{j}$, the natural logarithm equals $\;0\left(\;\ln(V({\vec{y}}_{j}\right)\to 0)$. Therefore, as the model becomes more certain, its learning ability becomes limited. The use of the weighted cross entropy loss function, $WM_{\left(\vec{y},\vec{w},\vec{z}\right)}$, is beneficial when some categories are more important than others or require more attention due to instruction. Two variables were used in the current study. The first one, $\vec{w}$, remained constant while considering the population size, and the second one changed $\vec{w}$ after each iteration based on the proportion of the category error, as calculated by the following formula.

(12) $$Mr_{t,j}=1-TV_{j,t}$$

The $Mr_{t,j}$ and $TV_{j,t}$ are used to show the ratio of the category error and actual positive ratio. Meanwhile, t and j are used to represent the number of iteration and the classes, respectively. When considering a specific category, the error proportion is equivalent to the sum of the incorrect negatives. An alteration utilizing α is used for every $Mr_{j.t}$, adding implication to the excellent error ratios. Eq. (13) was used to establish the weights of the class.

(13) $$w_{j,t}=\frac{\alpha \left(Mr_{j,t}\right)}{\sum_{i}^{4}\alpha \left(Mr_{i,t}\right)}$$

The recognition function displays a clear connection between the weight and the error ratio, as α(y) equals y. When α(y) is equal to e, raised to the power of y, the softmax function will yield the output.

The current investigation involved structuring α(y) which includes e in the $10y,y^{3},e^{y}$ and $e^{100y}$. The loss function established crucial categories that were emphasized with each iteration of the process. A slower pace of progress combined with an extended timeframe of instruction can yield greater efficiency. The training ratio utilized in this case was $10^{-5}$, which is comparatively smaller than the frequent training that utilized $10^{-4}$.

The focal loss function $\left(WM_{\vec{z},\vec{w},\vec{y}}\right)$ is more detailed and specific than the loss function of the weighted cross entropy $\left(WM_{\vec{z},\vec{w},\vec{y}}\right)$. It is composed of small granules that emphasize on specific details. $R_{\vec{z},\vec{y}}$ assigns weights to each training instance without considering its fit in a certain class or organization. In the current research, an improved model of the enhanced pelican optimization algorithm was utilized to minimize loss function by ensuring a well-organized network structure.

## 5 Dynamic pelican optimization algorithm

At first, the arrangement of the population-based pelican optimization algorithm and its mathematical modeling are examined.

### 5.1 Pelican behavior and motivation during hunting

Pelicans have a unique method of catching and swallowing their prey utilizing a significant bag in their pharynx. These social creatures prefer to live in groups, often consisting of several hundred individuals. While they do not typically target turtles, frogs, or crustaceans, pelicans regularly feed on fish. Hunting prey by pelicans usually takes place in groups with the participation of members. Their hunting method is that they first identify the prey and land on it from a height of 10–20 m. On the other hand, there is another type of pelican that attacks the target from the above. Once the pelican has landed, it opens its wings at the level of the water, which causes the fish to move towards shallower water, making them easier to catch. After catching the fish, the pelicans bend their heads forward to remove any excess water that may have entered their throats during the hunt, before consuming their meal.

According to these algorithms, pelicans are skilled and forceful hunters, and the descriptive method has been used in presenting proposed methods.

### 5.2 The proposed POA mathematical design

This algorithm recommends it describes each of the pelicans that make up the population in the algorithm.

The variable values in the optimization problem are defined and checked based on the location of every candidate in the environment and solution space. The way of forming the population of people based on the lower and upper limit of the issue is accidently determined in Eq. (14).

(14) $$k_{j,i}=b_{j}+rand.\;\left(a_{j}-b_{j}\right),i=1,2,\ldots ,N\;j=1,2,\ldots ,d\text{,}$$

In this equation, $k_{j,i}$ represents the $j^{th}$ value of the variable that can be determined by the $i^{th}$ individual, which is a separate solution. The number of population members is denoted by N, and the number of variables in the drawback is indicated by d.

To clarify, rand is used to define a random amount between 0 and 1, while $b_{j}$ and $a_{j}$ describe the lower and upper limit of the $j^{th}$ problem's parameters. As for the proposed POA, the population matrix provided below defines the separation of the pelican population.

Each of the responses is determined one by one by the variables of the issue, in turn, by the rows and columns of the matrix as defined in Eq. (15).

(15) $$K={\left[\;\begin{array}{c} k_{1}\\ \vdots \\ \begin{array}{c} k_{i}\\ \begin{array}{c} \vdots \\ k_{n} \end{array} \end{array} \end{array}\right]}_{N\times d}={\left[\begin{array}{ccc} k_{1,1} & \cdots & \begin{array}{cc} k_{1,j} & \begin{array}{cc} \cdots & k_{1,d} \end{array} \end{array}\\ \vdots & \ddots & \begin{array}{cc} \vdots & \begin{array}{cc} \vdots & \vdots \end{array} \end{array}\\ \begin{array}{c} k_{i,1}\\ \begin{array}{c} \vdots \\ k_{N,1} \end{array} \end{array} & \begin{array}{c} \cdots \\ \begin{array}{c} \vdots \\ \cdots \end{array} \end{array} & \begin{array}{cc} \begin{array}{c} k_{i,j}\\ \begin{array}{c} \vdots \\ k_{N,j} \end{array} \end{array} & \begin{array}{cc} \begin{array}{c} \cdots \\ \begin{array}{c} \ddots \\ \cdots \end{array} \end{array} & \begin{array}{c} k_{i,d}\\ \begin{array}{c} \vdots \\ k_{N,d} \end{array} \end{array} \end{array} \end{array} \end{array}\;\right]}_{N\times d}$$

The symbols K and $k_{i}$ refer to the matrix of the population of pelicans and the $i^{th}$ pelican at time. By utilizing the candidate solutions that were previously identified, the fitness function of the particular issue can be guessed. Eq. (16) shows the fitness function vector of the subordinate fitness.

(16) $$A={\left[\begin{array}{c} A_{1}\\ \vdots \\ \begin{array}{c} A_{i}\\ \begin{array}{c} \vdots \\ A_{N} \end{array} \end{array} \end{array}\right]}_{N\times 1}={\left[\begin{array}{c} A\left(K_{1}\right)\\ \vdots \\ \begin{array}{c} A\left(K_{i}\right)\\ \begin{array}{c} \vdots \\ A\left(K_{n}\right) \end{array} \end{array} \end{array}\right]}_{N\times 1}$$

The value of the proportionality function i is designate by $F_{i}$, which is a candidate function.

The solutions are updated by simulating pelicans' hunting behavior. The simulation stages include two parts, the first stage includes exploration (moving in the direction of hunting prey), and the second stage includes exploitation (moving and flapping in the direction of movement).

First step 1 Exploration (Movement in the direction of the prey)

Once the pelican has determined the hunting place, it begins to make its way towards it. This process can be simulated to demonstrate the algorithm's ability to search for solutions and explore various areas of the solution space. It is fascinating to see how this exploration capability can identify dissimilar zones and lead to valuable discoveries. In POA, the algorithm randomly creates a hunting place in the solution space, improving exploration ability of the solutions. The mathematical equation provided for this problem is represented in Eq. (17).

(17) $$k_{j,i}^{p1}=\left\{ \begin{array}{c} k_{j,i}+rand.\;\left(\;p_{j}-I.k_{j,i}\right),F_{p}<F_{i}\\ k_{j,i}+rand.\;\left(k_{j,i}-p_{j}\right),else\; \end{array}\right.$$

This equation shows that $k_{j,i}^{P1}$ represents the location of $i^{th}$ pelicans in the $j^{th}$ dimension in this step, I is a random value, like the number 3 and 4, which is chosen for each separate individual in each iteration, $F_{p}$ displays the value of the objective function and performance, $P_{j}$ shows the location of the prey in the $j^{th}$ dimension.

If the specified number of I is 2, it causes a member to move more, and it has the ability to investigate unknown places in space. In addition, the analysis of the solution space limits the effect of the $I^{th}$ solution.

When the fitness subordinate value is improved, the effectiveness and acceptance of the pelican's location improvement and new position are evident. This approach ensures that the algorithm does not target non-optimal areas. The mathematical expression of this method is provided in Eq. (18).

(18) $$K_{i}=\left\{ \begin{array}{c} K_{i}^{P1},F_{i}^{P1}\;<F_{i};\\ K_{i},else, \end{array}\right.$$

The new state of Pelican is defined as $K_{i}^{P1}$, and the value of the fitness function $F_{i}^{P1}$ is shown in step 1.

Second step 2 Exploitation (Winging on the plane of the water)

After landing on the water, pelicans extend their wings to direct fish into their pharynx. This hunting pattern allows pelicans to attack and catch a huge number of fish.

By simulating the behavior of pelicans, they have been able to improve pelicans' hunting techniques in the POA area. This has led to better results in terms of finding more optimal points. Not only has this improved Pelican's local search capabilities, but also it has increased the exploitation ability of the algorithm to its fullest potential.

From a mathematical perspective, checking the neighboring points of the Pelican position is necessary to converge to a better solution. According to these behaviors, Eq. (19) can be used.

(19) $$K_{j,i}^{P2}=k_{j,i}+R.\left(1-\frac{t}{T}\right).\;\left(\;2.\;rand-1\right).\;K_{j,i}\text{,}$$

here, $k_{j,i}^{P2}$ illustrates the new position of the pelican i within the dimension j agreeing to this step, R is a constant value the same as 0.2, $R.\left(1-\frac{t}{T}\right)$ demonstrates the radius of neighborhood $K_{j,i}$, t is the number of iterations, T represents the maximum amount of iterations that can occur.

The ability to use POA for achieving the global optimal solution is affected by the coefficient R, which is equal to $R.\left(\;1-\;\frac{t}{T}\right)$. This coefficient declines as the algorithm iterates; consequently, the neighborhood radius declines as well. The utilization of this coefficient provides an extra detailed examination of the area around every candidate, allowing POA to converge to global optimal solutions.

The reason for presenting Eq. (20) is to show whether or not to reject the new position of pelicans.

(20) $$K_{i}=\left\{ \begin{array}{c} K_{i}^{P2},F_{i}^{P2}\;<F_{i},\\ K_{i},else, \end{array}\right.$$

here, $K_{i}^{P2}$ illustrates the new location of pelican i, and $F_{i}^{P2}$ illustrates the benchmark function values based on the stage 2.

After renewing all the members of the candidate based on the steps outlined earlier, the best candidate solution is updated, using the new position of the individuals and their respective fitness function values. This process involves iteration of steps and the use of pseudo-code.

The algorithm repeats different stages based on the equations above. The quasi-optimal solution obtained through iterations is presented as the POA pseudo-code below.

The algorithm two has been described in the following. The optimization issues should be entered, and the number of iterations, and the candidate size N are determined in POA. The position of pelicans should be initialized, and fitness functions should be computed. When t equals 1, they should accidently make the position of the prey. First, it is necessary to act according to the first stage of exploration (movement in the direction of the prey), where the equation of I is equal to 1:N. After that, to calculate the dimension j, which is equal to 1:m, the location of the $j^{th}$ dimension can be obtained using Eq. (17), and in this case, the stage finishes. Then, Eq. (18) can be used to check and obtain the singular member of j. The exploitation part is conducted, in which j is equal to 1. Finally, Eq. (19) can be used to calculate the dimension j in the new location. Therefore, Eq. (20) can be used to renew members; eventually, the phase ends. Finally, according to the above criteria, the best single solution can be accomplished.

### 5.3 Dynamic POA (DPOA)

The Pelican Optimization Algorithm is a metaheuristic that emulates the behavior of pelicans in their natural habitat to optimize solutions. Nevertheless, the original algorithm may have certain limitations in terms of exploration and exploitation capabilities or convergence speed, contingent upon the problem domain. Consequently, modifications are imperative to augment its performance and make it more efficacious in resolving optimization problems. The present study introduces a new modification using fractions to improve precision and global applicability of this algorithm, addressing the limitations of the previous algorithm. In this study, the focus is on the term I. Instead of fixing the value of I to a specific number (such as 3 or 4), I should be regarded as a variable that changes dynamically in each iteration. One possible approach is to use a decreasing function of the number of iterations. Eq. (21) is an equation that incorporates this improvement:

(21) $$I_{i}=I_{\max}\times \exp\left(-\alpha \times \frac{i}{max_{iter}}\right)$$

where, $I_{i}$ represents the value of I for the current iteration i, $I_{\max}$ is the maximum value of I, α is a scaling factor that controls the rate of decrease, $max_{iter}$ is the total number of iterations.

Through the introduction of a dynamic scaling factor, the value of I undergoes a gradual reduction as the algorithm advances through successive iterations. This feature facilitates a greater capacity for exploration during the initial stages, while enabling a more targeted exploitation capability during the later stages of the optimization process.

## 6 Simulation results

### 6.1 System configuration

Owing to the random nature of the Dynamic Pelican Optimization Algorithm, the outcomes derived from its execution may exhibit variability across multiple runs. Consequently, a total of 20 iterations were conducted for each test. Furthermore, in addition to the Dynamic Pelican Optimization Algorithm, there existed several other algorithms that might be employed for the purpose of comparison, as discussed in the preceding section. All algorithms were executed with a maximum of 120 iterations and 20 runs, resulting in a total of 2400 function computations in order to ensure a fair comparison. The simulations were performed in the MATLAB R2018b environment, utilizing an Intel® Core™ i7-9700K @3.60 GHz 2, with 64 GB memory, and Windows 10 Professional 64-bit operating system.

**Fig. 6:**
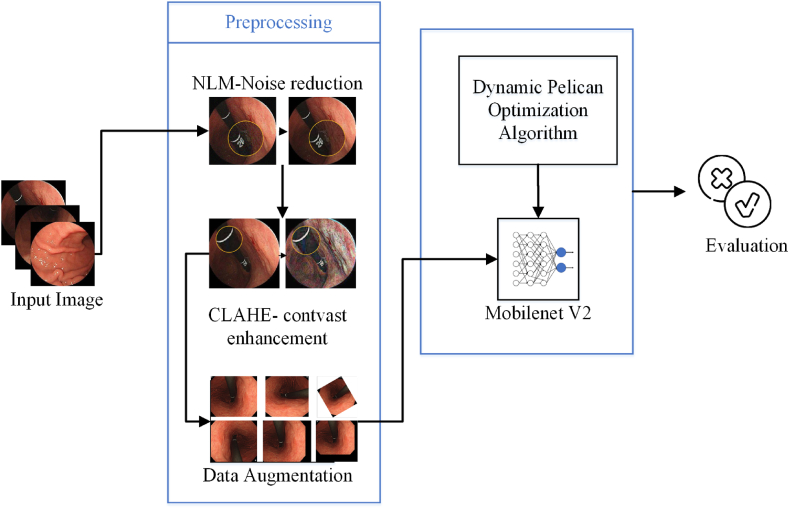
The graphical diagram of the proposed methodology.

The MobileNetV2/DPOA was trained on 80 % of the images from the Gastric images dataset, while the remaining 20 % was reserved for testing the model. The proposed methodology entailed as sequential steps as shown in Fig. (6).

**Fig. 7:**
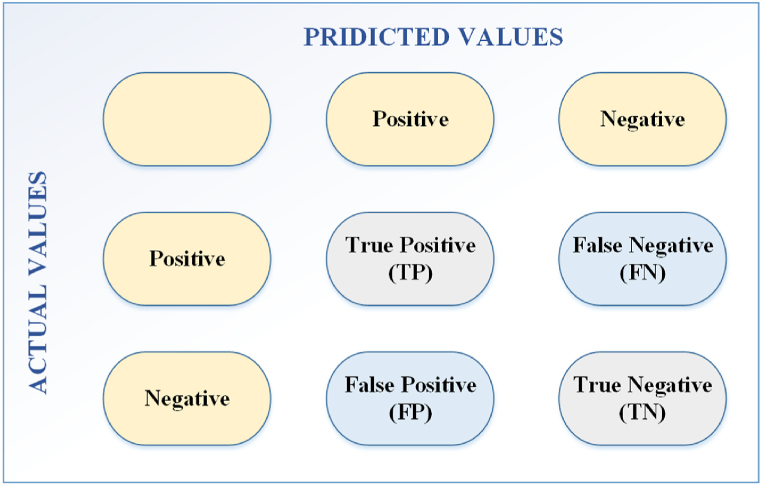
Confusion matrix of the evaluated indices.

The validity of the proposed method was confirmed through the use of performance metrics. In the context of Gastric pathology images, the fractions of True Negative (TN), True Positive (TP), False Negative (FN), and False Positive (FP) were defined by the number of pixels. Fig. (7) depicts the confusion matrix of the evaluated indices.

### 6.2 Algorithm authentication

As mentioned before, the “Dynamic Pelican Optimization Algorithm” is a modified version of the original algorithm that incorporated dynamic changes to the term I for improved exploration and exploitation capabilities. The specific modifications to the algorithm can be summarized as follows:

The proposed Dynamic Pelican Optimization Algorithm (DPOA) was subjected to scrutiny using 10 prevalent cost functions from the “CEC-BC-2017 test suite”, which encompassed both multimodal and unimodal fundamental problems. The primary objective was to minimize all 10 cost functions to the greatest extent possible. As a result, the algorithm that achieved the lowest value for each cost function demonstrated superior efficacy compared to its counterparts. The results of the proposed approach were analyzed and compared with other contemporary algorithms to evaluate their efficiency. The aforementioned algorithms comprised Tunicate Swarm Algorithm (TSA) [16], Gravitational Search Algorithm (GSA) [17], Owl Search Algorithm (OSA) [18], and Pigeon-Inspired Optimization Algorithm (PIO) [19]. Table 1 displays the parameter values set for the evaluated algorithms.

**Table 1:** Parameter values of the evaluated algorithms.

TSA [16]	Search agents	40
	$P_{\min}$	2
	$P_{\max}$	3
	Number of generations	120
GSA [17]	Search agents	40
	Gravitational constant	80
	Alpha coefficient	20
	Number of generations	120
OSA [18]	$T_{dead}$	20
	|P|	10
	$Acc_{low}$	0.2
	$Acc_{high}$	1
PIO [19]	Number of Pigeons	120
	Space dimension	20
	Map and compass factor	0.2
	Map and compass operation limit	110
	Landmark operation limit	100
	Inertia factor (w)	1
	Self-confidence factor ($c_{1}$)	1.1
	Swarm confidence factor ($c_{2}$)	1.1

Each optimizer has undergone 20 iterations on every function to validate the efficacy of the algorithms. The standard deviation and mean values were verified to produce consistent results across the iterations. The comparison results of the cost function between the DPOA and other tested optimizers are presented in Table 2.

**Table 2:** The comparison results of the cost function between the DPOA and other tested optimizers.

Benchmark		DPOA	TSA [16]	GSA [17]	OSA [18]	PIO [19]
f1	AVG	0.00	13.05	9.76	9.42	12.52
	STD	0.00	10.98	4.41	9.16	12.58
f2	AVG	0.79	9.54	15.25	13.84	12.90
	STD	0.55	14.63	12.63	6.95	7.92
f3	AVG	0.00	0.02	0.04	0.00	0.00
	STD	0.00	0.00	0.00	0.00	0.00
f4	AVG	0.00	0.00	0.00	0.00	0.00
	STD	0.00	0.00	0.00	0.00	0.00
F5	AVG	0.00	6.62	5.89	6.38	9.28
	STD	0.00	6.02	2.94	3.35	2.53
F6	AVG	0.19	10.58	8.17	9.44	8.50
	STD	0.17	4.85	3.55	5.01	3.56
F7	AVG	0.22	17.12	18.09	20.88	13.01
	STD	0.08	18.46	9.76	14.06	12.09
F8	AVG	0.00	2.32	1.11	1.96	2.22
	STD	0.00	1.29	1.53	0.80	3.10
F9	AVG	0.00	0.00	4.62	7.35	8.62
	STD	0.00	0.00	2.57	4.24	3.45
F10	AVG	0.00	9.49	2.80	5.89	6.42
	STD	0.00	7.37	1.95	2.11	2.60

The results of the comparison presented in Table 2 reveal that the Dynamic Pelican Optimization Algorithm (DPOA) consistently outperforms the other algorithms tested, as evidenced by both the average and standard deviation values. Specifically, DPOA achieved significantly lower cost function values on average compared to the other algorithms. For instance, for function 1, DPOA attained an average value of 0.00, while the other algorithms exhibited average values ranging from 9.42 to 13.05. This indicated that DPOA is more expert at identifying the global optimum and minimizing the cost function. Moreover, for functions 3 and 4, DPOA again achieved an average value of 0.00, indicating its ability to effectively reach the optimal solution, whereas the other algorithms failed to do so.

In terms of standard deviation values, DPOA consistently demonstrated lower variability than the other algorithms. A lower standard deviation implied that the algorithm produced more consistent and reliable results across multiple iterations. This underscores the stability and robustness of DPOA in consistently finding near optimal solutions. Overall, the combination of low average and standard deviation values of DPOA underscored its superior performance in terms of accuracy, precision, and stability when compared to the other optimization algorithms that were tested. These findings highlighted the efficacy of incorporating dynamic changes to the term I in DPOA, which enhanced its exploration and exploitation capabilities and enabled it to handle both multimodal and unimodal fundamental problems more efficiently.

### 6.3 Measurement indicators

In order to evaluate specificity, sensitivity, F1-score, accuracy, precision, and MCC (Matthew's correlation coefficient) of the automated Gastric cancer detection system, the several evaluation metrics were utilized, including sensitivity, particularity, accuracy, MCC (Matthew's correlation coefficient), and precision. In the following, the mathematical formulation of the indicators are given in Eq. (22) to Eq. (27).

(22) $$Specificity=\frac{TN}{TN+FP}\times 100$$

(23) $$Sensitivity=\frac{TP}{TP+FN}\times 100$$

(24) $$Accuracy=\frac{TP+TN}{TP+TN+FP+FN}\times 100$$

(25) $$Precision=\frac{TP}{TP+FP}\times 100$$

(26) $$MCC=\frac{TP\times TN-TP\times FN}{\sqrt{\left(TP+FP\right)\times \left(TP+FN\right)\times \left(TN+FP\right)\times \left(TN+FN\right)}}\times 100$$

(27) $$F1-score=2\times \frac{Precision\times Sensitivity}{Precision+Sensitivity}\times 100$$

### 6.4 Comparison analysis

The efficacy of the proposed MobileNetV2/DPOA model has been validated through the aforementioned metrics and compared with several previously published techniques, including Convolutional Neural Network (CNN) [3], Mask R–CNN [5], U-Net [6], deep stacked Sparse Autoencoder Neural Network (SANN) [7], and Deeplab v3+ neural network (Deeplab v3+) [8].

Table 3 presents the comparison results of the proosed MobileNetV2/DPOA model with other previously published methodologies.

**Table 3:** Comparison results of the proosed MobileNetV2/DPOA model with other previously published methodologies.

Technique	Accuracy	Precision	Specificity	F1-score	Sensitivity	MCC
CNN [3]	68.75	85.98	88.28	85.00	60.97	58.82
u-net [6]	78.15	93.29	92.75	85.13	65.35	65.54
SANN [7]	83.60	92.98	90.57	88.90	69.72	67.36
R–CNN [5]	86.50	89.48	90.92	91.58	78.35	69.85
Deeplab v3+ [8]	93.46	96.70	95.19	95.44	89.26	84.32
MobileNetV2/DPOA	97.73	97.88	97.72	98.41	96.35	96.58

**Fig. 8:**
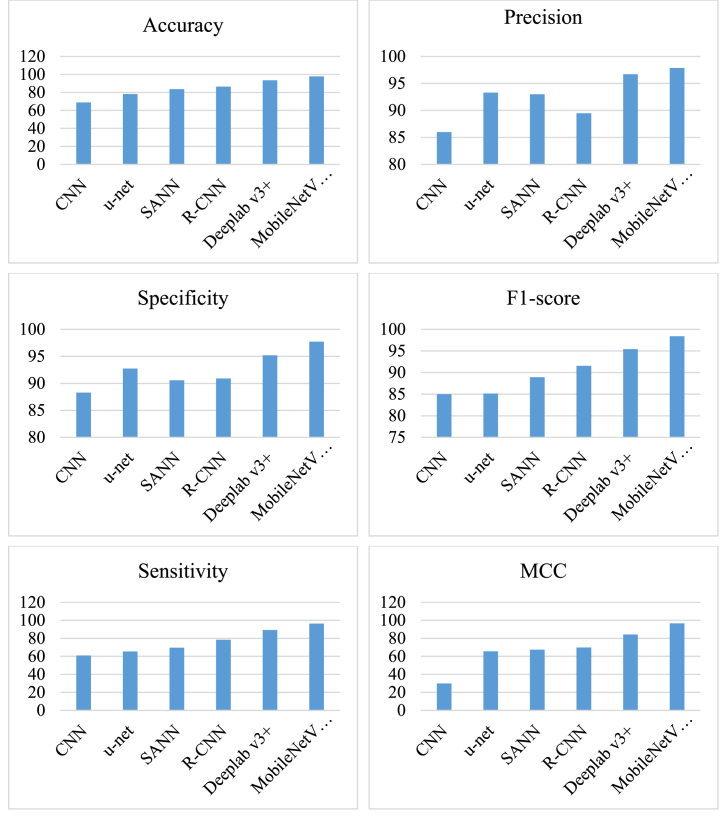
Graphical comparison results for the different methods.

Fig. (8) illustrates the graphical comparison results of the different methods.

As can be observed from Table 3 and Fig. (8), in terms of accuracy, the MobileNetV2/DPOA model achieved a remarkable accuracy of 97.73 %, outperforming all the other methods. This indicated the high ability of the MobileNetV2/DPOA model in correctly classifying gastric cancerous cases. Precision, which measures the proportion of correctly predicted positive cases among all predicted positive cases, of the MobileNetV2/DPOA model reached 97.88 %. It demonstrated the model's strong capability to minimize false positives.

Specificity, representing the percentage of correctly classified negative cases among all actual negative cases, yielded a value of 97.72 % for the MobileNetV2/DPOA model, indicating its effectiveness in correctly identifying non-cancerous cases. The F1-score, which considers both precision and sensitivity, serves as an overall measure of the model's performance. The MobileNetV2/DPOA model achieved an impressive F1-score of 98.41 %, signifying its ability to achieve a balance between precision and sensitivity.

Sensitivity, also known as the true positive rate, measures the proportion of correctly classified positive cases among all actual positive cases. The MobileNetV2/DPOA model demonstrated a sensitivity of 96.35 %, indicating its proficiency in accurately detecting gastric cancerous cases. Furthermore, the MCC (Matthew's correlation coefficient) of the MobileNetV2/DPOA model reached 96.58 %. The MCC took into account true positive, true negative, false positive, and false negative values, providing a comprehensive assessment of the model's performance. Comparing these metrics with the other methodologies, it is evident that the MobileNetV2/DPOA model outperformed all the previously published techniques in terms of accuracy, precision, specificity, F1-score, sensitivity, and MCC. These results highlighted the efficacy and superiority of the proposed MobileNetV2/DPOA model for automated gastric cancer detection.

## 7 Conclusions

The presented work in this study proposed a novel methodology for the early diagnosis of gastric cancer, utilizing the MobileNetV2 model that were enhanced by a new design of Pelican optimization algorithm, called Dynamic Pelican Optimization Algorithm (DPOA). By applying this methodology, the researchers were able to surpass the performance of existing approaches in the field, thus showcasing its potential impact on improving patient outcomes. To analyze the method's efficiency, it was applied to a standard benchmark, and its results were compared with some other state-of-the-art methodologies, including Convolutional Neural Network (CNN), Mask R–CNN, U-Net, deep stacked Sparse Autoencoder Neural Network (SANN), and Deeplab v3+ Neural Network (Deeplab v3+) to solidify its efficacy and practicality in real-world clinical settings. The results showed that the proposed MobileNetV2/DPOA model demonstrated exceptional performance in detecting gastric cancer compared to other popular models. It has achieved an impressive accuracy of 97.73 %, precision of 97.88 %, specificity of 97.72 %, F1-score of 98.41 %, and sensitivity of 96.35 %. These statistics highlighted the advantages of incorporating the dynamic optimization algorithm with MobileNetV2 for gastric cancer detection. However, it is important to acknowledge certain limitations in the current research. Firstly, the small sample size of gastric images raises concerns about the generalizability of the results. Future efforts should prioritize obtaining larger and more diverse datasets for comprehensive analysis. Secondly, despite the compelling performance metrics, it is crucial to subject the model to rigorous clinical validation. Employing feedback from experienced specialists can enhance the model's functionality and tailor it to meet real-world clinical requirements. Lastly, the absence of external testing calls for collaborative efforts among multiple centers to verify the model's robustness and reliability. Engaging with international partners will ensure its credibility and readiness for widespread adoption. By expanding datasets, considering expert opinions, and conducting external tests, the innovative MobileNetV2/DPOA model holds great promise in advancing automated gastric cancer detection, ultimately transforming patient care and treatment decisions.

## Data availability statement

The research presented in this paper utilizes the “Japanese classification of gastric carcinoma” dataset, which can be obtained by sending an email request to *jgca@koto.kpu-m.ac.jp*.

## CRediT authorship contribution statement

**Guoping Zhou:** Formal analysis, Data curation, Conceptualization. **Qiyu He:** Formal analysis, Data curation, Conceptualization. **Xiaoli Liu:** Formal analysis, Data curation, Conceptualization. **Xinghua Kai:** Formal analysis, Data curation, Conceptualization. **Weikang Cao:** Formal analysis, Data curation, Conceptualization. **Junning Ding:** Formal analysis, Data curation, Conceptualization. **Bufeng Zhuang:** Formal analysis, Data curation, Conceptualization. **Shuhua Xu:** Formal analysis, Data curation, Conceptualization. **Myo Thwin:** Formal analysis, Data curation, Conceptualization.

## Declaration of competing interest

The authors declare that they have no known competing financial interests or personal relationships that could have appeared to influence the work reported in this paper.
